# Segmented socioeconomic adaptation of New Eastern European professionals in the United States

**DOI:** 10.1186/s40878-018-0077-3

**Published:** 2018-04-17

**Authors:** Nina Michalikova

**Affiliations:** Department of Sociology, Gerontology and Substance Abuse Studies, University of Central Oklahoma, 100 North University Dr. Edmond, OK, 73034 USA

**Keywords:** Socioeconomic adaptation, Post-1991 Eastern European professionals, Education, Occupation, Income

## Abstract

This study examines the socioeconomic adaptation of post-1991 Eastern European professionals in the United States. The data were obtained from the pooled 2006–2010 American Community Surveys. The analysis includes recent immigrants between ages of 25–65 who have at least an associate’s degree. Skilled immigrants in professional or managerial occupations are compared with non-professionals or managers to examine and compare socioeconomic outcomes. The findings presented in this study support the segmented assimilation theory and reveal cross-group and cross-country disparities in socioeconomic adaptation. Despite the high amount of human capital, Eastern European skilled immigrants tend to have a lower share of professionals and managers than other groups. Their average income is lower than the income of some other groups in the analysis, especially immigrants from Northern and Western Europe, suggesting these immigrants experience difficulties in transferring human capital. Among the three largest Eastern European groups – Russia, Ukraine, and Poland – there is a clear hierarchy in socioeconomic status with Russian professionals having the highest educational attainment and income, followed by immigrants from Ukraine and Poland. Results also revealed gender differences in socioeconomic adaptation. Women from Eastern Europe are highly professional, but they tend to be concentrated in different occupations than men, leading to a significant gender-wage gap. The effect of selected individual and country-level characteristics on skilled immigrants’ socioeconomic adaptation is discussed.

## Introduction

In 2015, nearly one-third of foreign-born workers in the United States were employed in professional, management, or related occupations (Bureau of Labor Statistics, 2016). The majority of U.S. foreign-born skilled workers originated in Asia (India, China, Philippines and South Korea), Western Europe (United Kingdom, France, and Germany), Canada, and Mexico (Department of Homeland Security, 2015), but in the past two decades an important influx of skilled foreign-born laborers appeared from Eastern Europe. Following the fall of the Soviet Union, many highly educated and professional migrants left Eastern Europe to pursue further training or to search for better career opportunities in the U.S. (Portes & Rumbaut, 2014). Between 2003 and 2016, the period for which data is available, 98,034 professional immigrants and managers from Eastern Europe were admitted into permanent residency in the United States. Most of these immigrants originated in Russia (21,759), Poland (13,364) and Ukraine (19,168) (Yearbook of Immigration Statistics, 2016).

Prior research examining adaptation experiences of Eastern European skilled immigrants in the U.S. is limited. Existing studies show that new Eastern European immigrants have similar educational attainment than immigrants from Western Europe and other developed countries (e.g., Canada, Australia, and Japan); also, Eastern European college degree holders have lower chance of obtaining a skilled occupation ranging between 35% for Polish immigrants and 55% for Hungarian immigrants (Mattoo, Neagu, & Özden, 2008). This suggests that Eastern Europeans tend to have a lower degree of skill transferability than immigrants from English-speaking countries and Japan (Chiswick & Taengnoi, 2007; Haley & Taengnoi, 2011).

Why do new Eastern European immigrants with high amounts of human capital lag behind other equally educated immigrant and professional groups in their occupational attainment and income? An examination of socioeconomic backgrounds of Eastern European professionals will help to explain the discrepancy and fill the gap in the literature on this under-studied immigrant population and on immigrant professionals in the U.S. in general. This is the first study to examine how post-1991 Eastern European professionals adapt socioeconomically in the U.S. and how selected individual and country-level characteristics affect their socioeconomic adaptation. A systematic examination of these new immigrant professionals and their occupational and income patterns extends the limited scholarship on immigrant professionals in the U.S. and suggests that policymakers create new or revise existing policies (Haley & Taengnoi, 2011).

Adaptation refers to the performance of immigrants in the host country. This study focuses on socioeconomic adaptation, which is the adjustment of immigrants to life in their host country through socioeconomic attainment, and it is a major determinant of the immigrants’ overall well-being. Socioeconomic attainment indicates “the possession of scarce economic resources and social characteristics that are valued in society” (Sakamoto & Xie, 2006, p. 54). Successful socioeconomic adaptation facilitates immigrants’ integration into their communities, defined as “the process of economic mobility and social inclusion for newcomers and their children” (Migration Policy Institute, 2018).

The next sections discuss the backgrounds of immigrant professionals in the U.S., followed by the overview of post-1991 Eastern European skilled immigrants, their characteristics, and determinants of their socioeconomic adaptation.

## Immigrant professionals in the United States

Since the 1990s, international migration of professionals has been increasing (Slade, 2015), but the experiences of skilled workers have not been systematically studied and the literature on professionals migrating internationally is scarce compared to studies examining challenges faced by low-skilled migrants (Remennick, 2003).

Prior studies described the economic integration process of immigrant professionals as a complex inter-play of factors that determine if human capital will be converted into economic success (Schittenhelm & Schmidtke, 2011). The barriers most commonly experienced by immigrant professionals include non-recognition of foreign credentials, devaluation of international work experience, limited opportunities to gain appropriate work experience, discrimination, lack of professional networks, difficulties adopting a new culture, language barriers, or cumbersome and costly licensing (Gauthier, 2016; Schittenhelm & Schmidtke, 2011; Slade, 2015). These experiences suggest that while many developed countries implement policies to attract skilled workers, non-recognition of foreign credentials and prior work experience in immigrant-receiving countries reduces the immigrants’ earnings and contributes to their downward social mobility (Guo, 2009).

The U.S. remains one of the main destinations for skilled immigrants. Due to increasing globalization and demographic changes, the demands for skilled professional immigrants will likely continue in the future (Facchini & Lodigiani, 2014). In 2014, about 31% of employed immigrants held professional, managerial, or related occupations, compared to 40% of native-born workers who were professionals or managers (Bureau of Labor Statistics, 2016). Among newly admitted lawful permanent residents, 12% held a managerial, professional, or related occupation, and 24% of new lawful permanent residents obtained their green cards based on employment preferences (Yearbook of Immigration Statistics, 2016). Of all employment-sponsored immigrants, 36% were professionals with advanced degrees or immigrants of exceptional ability. Since 2004, the percentage of lawful permanent residents who received their green cards through employment fluctuated between 15 and 30%, and between 17 and 52% of new employment-based green card holders were professionals with advanced degrees or immigrants of exceptional ability (Yearbook of Immigration Statistics, 2014). These statistics indicate that skilled workers have played a crucial role in the building of the U.S. economy and achieving U.S. international competitiveness (Jasso, 2009), but very few studies examined their socioeconomic status and experiences in the U.S. labor market (Haley & Taengnoi, 2011).

The numbers of Eastern European immigrants in the United States increased following the fall of communism in Eastern Europe and the collapse of the Soviet Union in 1991 (Table 1). The data from the Yearbook of Immigration Statistics (2012) show that 1,523,565 new Eastern European immigrants were admitted to permanent residency during the 1991–2012 period, twice the number of Eastern European immigrants admitted during the Cold War era, which lasted almost fifty years (Yearbook of Immigration Statistics, 2012). During this period, the largest Eastern European immigrant groups in the U.S. were Ukrainians (306,584), Polish (299,548), Russians (285,764), Bosnian and Herzegovinians (129,868), and Romanians (118,812).

**Table 1:** Trends in Eastern European immigration by country of birth, 1991–2012

Country	1945–1990	1991–1995	1996–2000	2001–2005	2006–2012	1991–2012
Albania	4068	5133	21,058	21,272	36,229	83,697
Belarus^a^	X	17,146	11,844	13,440	15,872	58,310
Bosnia and Herzegovina^a^	X	4756	34,373	79,704	10,989	129,868
Bulgaria	8326	5907	17,672	21,721	22,573	67,456
Croatia^a^	X	1467	3741	11,095	3420	19,732
Czech Republic/Slovakia^b^	90,661	83	884	1774	12,538	33,435
Hungary	113,727	5709	4664	6397	8531	25,311
Latvia	42,464	2631	2717	3225	3656	12,185
Lithuania	33,085	2536	5586	10,681	8127	26,830
Macedonia^c^	X	1033	3796	4240	7726	16,798
Moldova^a^	X	8467	5785	10,335	14,639	39,235
Poland	441,026	114,421	55,191	64,667	65,320	299,548
Romania	95,421	28,512	29,023	26,844	34,412	118,812
Russia^a^	X	50,745	77,286	90,484	67,149	285,764
Ukraine^a^	X	71,141	70,156	90,638	74,588	306,584
Total	828,778	324,449	348,889	464,458	385,769	1,523,565

Source: 2012 Yearbook of Immigration Statistics

Data not available in some years before 1991 for Albania, Bulgaria, Latvia and Lithuania

^a^Not applicable before 1992

^b^Before 1993, Czechoslovakia

^c^Not applicable before 1994

An important characteristic of new Eastern European immigrants is their high level of educational attainment and professionalization (Portes & Rumbaut, 2014). The 2006–2009 American Community Surveys (ACS) data (U.S. Census Bureau, 2012) show that about 69% of post-1991 Eastern European immigrants had some higher education (including some college, bachelor’s degree, or advanced degree), with 22% possessing an advanced degrees beyond college (Table 2). Eastern Europeans were more likely to complete college or hold professional or graduate degrees (46%) than all foreign-born (30%), black immigrants (25%), and Hispanic immigrants (10%), but they lagged behind Asian immigrants (57%). Corresponding to their educational backgrounds, 37% of new Eastern European immigrants were professionals or managers and 53% were white-collar workers, including professionals or managers. Compared to other groups, Eastern Europeans had a higher likelihood of professionalization than all foreign-born (28%), black (26%), and Hispanic immigrants (11%), but they were less likely to hold professional or managerial occupations than Asian immigrants (51%) and the native-born (40%).

**Table 2:** Percentage distributions in education, occupational attainment, and personal income of post-1991 Eastern European immigrants and various foreign-born groups, 2006–2009 ACS

	All foreign-born	Eastern European	Asian	Black	Hispanic
Education					
Less than high school	30.6	6.0	12.4	17.5	50.7
High school	22.9	26.0	15.1	28.5	26.4
Associate’s degree	16.1	23.0	15.3	28.8	12.4
Bachelor’s degree	17.6	24.0	32.1	16.6	7.3
Advanced degree	12.6	22.0	25.3	8.6	3.2
N	570,755	34,823	159,526	43,255	253,265
N weighted	14,507,837	807,087	3,676,229	1,202,630	7,060,622
Occupation					
White-collar	42.6	53.0	68.3	46.3	22.9
Professional/Managerial	27.9	37.0	50.7	26.4	11.4
N	450,486	29,032	117,591	35,932	207,098
N weighted	11,637,371	679,363	2,720,638	1,009,229	5,875,577
Personal income					
Mean	35,735	35,900	46,413	32,936	24,740
$0 or less	4.6	5.0	5.2	3.9	4.5
$1- $9999	15.2	17.0	13.5	14.7	16.9
$10,000- $19,000	21.9	16.0	14.2	18.4	29.8
$20,000- $49,999	37.1	39.0	33.3	43.1	39
$50,000- $79,999	11.7	14.0	16.9	13.5	6.8
$80,000- $99,999	3.5	4.0	6.5	3.2	1.3
$100,000 or higher	5.9	5.0	10.4	3.2	1.7
N	868,431	28,741	230,647	67,237	390,352
N weighted	20,954,003	671,867	5,100,968	1,783,045	10,243,912

Considering their high amount of human capital, post-1991 immigrants from Eastern Europe do not fare very well in income. Based on the 2006–2009 ACS data, their average income was $35,900, about the same as the average income as all foreign-born ($35,735), somewhat higher than the income of foreign-born blacks ($32,936), but substantially lower than the income of foreign-born Asians ($46,413). Socioeconomic backgrounds of Eastern European immigrants suggest a discrepancy between educational attainment and occupational status and income, but it is unclear whether it persists at all educational levels and across occupational categories. Narrowing the focus of this study on experiences of skilled immigrants from Eastern Europe will shed some light on this prevalent and little understood phenomenon.

## Theory and hypotheses

Theories of immigrant adaptation can be applied to explain the socioeconomic adaptation of new Eastern European professionals. The classical assimilation model is one of the earliest explanations of socioeconomic adaptation of immigrants in the host society. According to this theory, all immigrants will experience upward socioeconomic mobility and they will gradually become inevitably and completely absorbed into the dominant Anglo-culture and institutions (Yang, 2000). If classical assimilation accurately explains socioeconomic integration of Eastern European professionals, than all professionals from this region should fare well socioeconomically with the passage of time. That is, over time, the immigrants’ high educational attainment should translate into corresponding occupational standing and income regardless of their country of origin.

In a bumpy-line assimilation model, Alba and Nee (2003) challenge the straight-line assimilation and argue that the assimilation process is uneven. Contemporary immigrants will eventually, inevitably assimilate, but different racial and ethnic groups may experience different rates of assimilation. According to this theory, all Eastern European professionals will adapt socioeconomically, but the extent of their adaptation may vary across groups.

Confronting the assumption that assimilation is inevitable for all groups, the segmented assimilation theory proposed by Portes and Zhou (1993) suggests that socioeconomic adaptation outcomes of contemporary immigrants are diverse. Immigrants may become integrated into any sector of American society resulting in one of the three possible assimilation outcomes: 1) upward mobility into white middle class; 2) downward mobility into an underclass; and 3) upward socioeconomic mobility, but delayed acculturation and intentional maintenance of immigrant cultures and institutions. Barriers to employment faced by immigrant professionals in the U.S. can lead to segmented socioeconomic outcomes. Prior research found that American immigrants have a higher likelihood of mismatch between educational attainment and occupational standing than the native-born (Beckhusen, Florax, Poot, & Waldorf, 2013). One form of such mismatch is over-education, which occurs when employees have more than the required level of education (Beckhusen et al., 2013). Unable to transfer their educational and professional experience, newcomers are often excluded from the labor market and end up working in jobs for which they are overqualified, resulting in “brain waste” (Schittenhelm & Schmidtke, 2011). As predicted by the segmented assimilation theory, socioeconomic trajectories of new Eastern European professionals may be diverse. These immigrants originate from a region with a shared history of communism, yet diversity in individual and country-level characteristics may contribute to segmented socioeconomic adaptation of different groups.

The literature identified several determinants of socioeconomic adaptation among immigrant professionals. Length of residency in the host country improves socioeconomic outcomes of skilled immigrants. With the passage of time, immigrants gain knowledge of the society, acquire country-specific skills, and are better equipped to compete for higher paying jobs (Beckhusen et al., 2013; Bodankin & Semyonov, 2016). Older professionals adapt better socioeconomically, as they have a higher amount of human capital and experience (Bodankin & Semyonov, 2016). Prior research found regional variations in socioeconomic outcomes, with immigrants residing in central cities having higher socioeconomic status than others (Bodankin & Semyonov, 2016). In the U.S., immigrant professionals tend to concentrate in “traditional” immigrant states including California, New York, Florida, and New Jersey (Batalova & Lowell, 2007). According to the U.S. Population Census, Northeast is a preferred region of residence among new immigrants from Eastern Europe (Grieco & Trevelyan, 2009) and residing in the region with higher concentration of co-ethnics may increase their level of socioeconomic adaptation. Education is a significant determinant of socioeconomic adaptation because having an advanced degree increases occupational status and decreases the likelihood of over-education (Chiswick, 1978). Among immigrants in Canada, for example, over-education reached 40% among professionals with bachelor’s degrees, 50% among doctoral/professional degree holders, and 75% among those with master’s degrees (Beckhusen et al., 2013). Being married is associated with better socioeconomic adaptation among immigrant professionals (Bodankin & Semyonov, 2016). Having a spouse present represents an attachment to the U.S. and may serve as an important source of social support and encouragement, contributing to better socioeconomic adaptation among married professionals (Constant & Massey, 2002). English proficiency is another individual predictor facilitating socioeconomic adaptation. Professionals who speak English well are less likely to experience over-education than immigrants with limited English language skills (Beckhusen et al., 2013).

Professional immigrant women are more likely to experience non-recognition of foreign credentials than men. They tend to have more family-related time constraints and prioritize their partner’s work, which likely limits their employment options. Their networks tend to be less varied and more family/community-oriented than networks of their male counterparts. Women are also more likely to experience gender-related prejudice and discrimination in the labor market than men, resulting in lower levels of socioeconomic adaptation (Aure, 2013; Gauthier, 2016; Guo, 2009).

A country of origin has been recognized as an important predictor of immigrants’ socioeconomic adaptation. Foreign-born professionals from English-speaking developed countries and Europe (excluding Eastern Europe) are able to transfer their skills more easily in the U.S. labor market than their respective counterparts, resulting in their representation in all skilled occupations (Haley & Taengnoi, 2011). The literature characterized immigrants from the former Soviet Union (FSU) as highly educated and professional, but despite their high amount of human capital, many had experienced downward social mobility (Bodankin & Semyonov, 2016). Based on GDP per capita, Moldova is the least economically developed FSU country in the analysis followed by Ukraine (The World Bank, 2014), however, because of the small sample size of respondents from Moldova, Ukraine was selected as the reference category for the country of origin variable in regression analyses to ensure the reliability of results. It is expected that professionals from other Eastern European countries in the analyses generally adapt better socioeconomically in the United States than Ukrainian immigrants.

Professionals from economically less developed countries and countries where political and personal freedoms are limited may experience more constraints to socioeconomic adaptation than immigrants from economically stronger countries. The literature suggests that immigrants originating from less developed countries are less able to transfer their skills than immigrants from countries that are similar to the host country in economic structures, technological advancement, or cultural orientation. In Israel, for example, professionals from European countries (other than the FSU) and North America were among the most successful groups and had better opportunities to find jobs that matched their educational level. This was explained by the resemblance between the economic and social structures of Israel and advanced Western countries and the immigrants’ membership in the dominant ethnic group in Israel (Bodankin & Semyonov, 2016). Among professionals in Canada, immigrant men from outside Europe earned 15–25% lower incomes than most of the other European groups, further supporting the pattern of diverse experiences depending on the immigrants’ origin (Reitz, 2001).

The demand for skilled labor is not unique to the United States. In recent decades, many developed countries have adopted policies to target skilled immigrants and facilitate their admission (Batalova & Lowell, 2007; Chiswick & Taengnoi, 2007). United States’ immigration laws provide a variety of ways for migrants to apply for permanent residency in the U.S. Immigration is possible under the following classes of admission: (1) family-sponsored preferences; (2) employment-based preferences; (3) immediate relative of U.S. citizens; (4) refuge or asylee; (5) Diversity Immigrant Visa Program; or under one of the several other categories (U.S. Citizenship and Immigration Services, 2017).

The admission of immigrants continues to be regulated by the 1960s legislation (Boyd, 2014). The 1965 Immigration Act remains the basis of the U.S. immigration policy and emphasizes family-sponsored immigration, not employment-based immigration (Boyd, 2014; Yang, 2000). Family-sponsored immigration does not require any specific skill or ability and family-sponsored immigrants are often not professionals. Immigrant professionals tend to be employment-based immigrants, so by emphasizing family-sponsored immigration and reunification, the current immigration policy decreases the share of immigrant professionals.

Skilled workers tend to enter the U.S. under one of the following employment-based visa categories depending on their educational background, skills, and abilities: (1) EB-1 includes foreign nationals with extraordinary ability in the sciences, arts, education, business, or athletics, outstanding professors and researchers, or certain multinational managers and executives; (2) EB-2 includes foreign nationals who are members of the professions holding advanced degrees or who have exceptional ability (including requests for national interest waivers); (3) EB-3 includes skilled workers, professionals, or other workers; (4) EB-4 includes religious workers and special immigrant juveniles; and (5) EB-5 includes immigrant investors. The first three preference categories require advanced education or exceptional abilities, and therefore, are the primary admission categories for immigrant professionals in the U.S. (U.S. Citizenship and Immigration Services, 2017).

## Data and methods

### Sample

The data were drawn from the pooled 2006–2010 American Community Surveys (ACS) collected by the U.S. Census Bureau (2012). Due to the differences in coding of the variable occupation across years, year 2010 could not be included in the analyses, so only years 2006–2009 were analyzed. The 2006–2010 ACSs were the latest datasets available when this project started and allow accurate comparisons with findings presented in earlier studies on post-1991 Eastern European immigrants. The ACS’s include many demographic, cultural, and socioeconomic variables and a large sample of skilled Eastern European immigrants to ensure reliable statistical analyses.

Definitions of skilled immigrants in the literature vary depending on the data, methodology, and goals. The term “skilled” is often used interchangeably with “professional” and “highly skilled” (Batalova & Lowell, 2007). The most obvious indicators of “skill” are education and occupation, which prior studies used to distinguish skilled immigrants from general immigrant population. This study uses a common international definition which classifies skilled immigrants as individuals who have completed a two-year college degree or more and are employed in a variety of skilled occupations (Batalova & Lowell, 2007). Based on this definition, new skilled immigrants from Eastern Europe are those who have completed an associate’s degree or higher and have been employed in a professional or managerial occupation based on the 2010 U.S. Census Occupational classification.

There are different definitions of Eastern Europe. Based on Robila (2010), this study adopts a geopolitical definition of Eastern Europe that formed the so-called “Communist bloc” during the “Cold War” where people shared the common experience of the “Iron Curtain.” Under this definition, Eastern Europe includes the following countries: Albania, Belarus, Bosnia and Herzegovina, Bulgaria, Czech Republic, Croatia, Estonia, Hungary, Latvia, Lithuania, Macedonia, Moldova, Poland, Romania, Serbia, Montenegro, Slovakia, Slovenia, Russia, and Ukraine.

Only the foreign-born Eastern European immigrants who arrived to the United States in or after 1991 were studied. This year marks the end of communist regimes in all of Eastern Europe resulting in the significant increase in the number of immigrants from this region (Table 1). The data were further restricted to adult respondents aged 25 to 65 because formal education is normally completed by the age of 25 and full retirement age in the U.S. is 66 or 67, depending on the birth year. No citizenship status restrictions were made and the experiences of both naturalized citizens and non-citizens were examined. The datasets did not include the information on the immigrants’ class of admission and whether they were admitted through a family-sponsored category or an employment-based category. Immigrants from Estonia, Slovenia, Serbia, and Montenegro were not included in the datasets.

There are three samples used in this study. The first sample (*N* = 8871) included post-1991 Eastern European immigrants aged 25 to 65 who completed an associate’s degree or higher and held a professional or managerial occupation. For comparison, the second sample (*N* = 5699) included post-1991 Eastern European immigrants aged 25 to 65 who completed at least an associate’s degree, but who did not hold a professional or managerial occupation, and were employed as other white-collar workers, service workers, manual workers, or unemployed. The third sample (*N* = 14,570) included all post-1991 Eastern European immigrants aged 25 to 65 with at least an associate’s degree, regardless of their occupational status.

### Variables and measurements

The study provides a socioeconomic profile of new Eastern European skilled immigrants by focusing on their education, occupation, and income. *Education* was an ordinal variable coded in the following dummy variables: (1) associate’s degree (degree awarded upon a completion of two years of study at a junior college, college, or university); (2) bachelor’s degree; (3) master’s degree; (4) professional degree (degree beyond a bachelor’s degree, including law, medical, veterinary, and dental degrees); (5) doctorate degree. The original variable measuring *occupational background* was recoded according to the 2010 Occupational Code List of the U.S. Census Bureau in five dummy variables: (1) managerial and professional occupations, (2) other white-collar workers, (3) service workers, (4) manual workers, (5) unemployed. The original variable *total person’s income* was recoded into an ordinal scale and was, also, converted to logarithm-based values.

The individual-level independent variables used as predictors of socioeconomic adaptation included the following: *length of stay* [a continuous variable that measures the number of years a respondent has lived in the U.S.]; *age* [measured in years]; *sex* [male = 1]; *marital status* [married, spouse present in the U.S. = 1]; *region of residence* [state of residence was coded in the following dummy variables: (1) Northeast, used as a reference category, (2) Midwest, (3) West, (4) South]; *survey year* [a series of dummy variables indicating when respondents were interviewed: (1) 2006, reference category, (2) 2007, (3) 2008, (4) 2009]; *English proficiency* [ordinal variable coded in the following categories: (1) Do not speak English, (2) Speak English, but not well, (3) Speak English well, (4) Speak English very well, (5) Speak only English]; and *self-employment* [self-employed = 1].

Two country-level variables were tested. The 2005 estimates of *GDP* per capita by the World Bank (2014) were used to create the variable measuring socioeconomic development in the immigrants’ countries of origin and it was coded as follows: (1) countries with a GDP per capita lower than $5000 (Romania, Bulgaria, Macedonia, Belarus, Bosnia and Herzegovina, Albania, Ukraine, and Moldova) and (2) countries with a GDP per capita of $5000 or higher (Czech Republic, Hungary, Croatia, Slovakia, Poland, Latvia, Lithuania, and Russia). The value $4954 is a median GDP per capita for all countries in the analysis; thus, $5000 is a meaningful cut-off point.

The extent of *political and personal freedom* in respondents’ countries of origin was determined based on the country rankings from the Freedom in the World, a survey created by Gastil (1980) and published by the Freedom House. The countries were ranked by political rights and civil liberties as free (Bulgaria, Croatia, Czech Republic, Hungary, Latvia, Lithuania, Poland, Romania, and Slovakia), partly free (Albania, Bosnia and Herzegovina, Macedonia, Moldova, and Ukraine), or unfree (Belarus and Russia). These rankings were used to create three dummy variables: (1) free countries (free countries = 1, used as a reference category); (2) partly free countries (partly free countries = 1); and (3) unfree countries (unfree countries = 1).

### Methods

The study used descriptive statistics to examine the socioeconomic status of Eastern European skilled immigrants, their individual backgrounds, and characteristics of their countries of origin. Regression analyses tested the determinants of occupational status and income among Eastern European skilled immigrants with an associate’s degree or higher. Logistic regression was used to assess the likelihood of holding a professional or managerial job because the dependent variable occupational status was dichotomous. The dependent variable income was a continuous measure; therefore, ordinary least squares regression (OLS) was the most appropriate regression technique to test the determinants of income.

## Findings

### Demographic characteristics

Table 3 shows that Eastern European immigrants with at least an associate’s degree who hold a professional or managerial occupation have been residing in the U.S. an average of 14 years, slightly longer than Asian, black, Hispanic, and other European immigrants. Eastern European immigrant professionals have a lower share of males (44%) than other groups. This gender difference may reflect accessibility of higher education in Eastern Europe to both genders. The average age of professionals from Eastern Europe is almost 39 years, slightly higher than the average age of other groups, except immigrants from Northern and Western Europe. The percentage of skilled immigrants from Eastern Europe who are married with their spouse present (70%) is comparable to Asian professionals (71%), but is higher than among other immigrant groups. Unlike other groups, Eastern European professionals are concentrated in the Northeast (36%). Almost one in three Eastern European skilled workers is from Russia (30%), followed by Ukraine (18%) and Poland (14%), which is understandable as these countries tend to send the highest number of Eastern European immigrants.

**Table 3:** Percentage distributions in selected immigrant characteristics of post-1991 immigrants with an associate’s degree or higher from Eastern Europe and various foreign-born groups, 2006–2009 ACS

	All EE		EE women		EE men		Polish	Russian	Ukrainian	N/W Europe	Asian	Black	Hispanic
Professionals/Managers	Yes	No	Yes	No	Yes	No	Yes	Yes	Yes	Yes	Yes	Yes	Yes
Individual-Level Variables													
Length of stay in the U.S.	13.5	12.5	13.4	12.3	13.6	12.7	13.3	13.9	14.4	10.9	11.6	12.7	11.8
Age (Mean)	38.8	41.4	38.4	40.7	39.3	42.3	36.5	40.5	40.3	39.0	36.4	37.7	38.2
Male	44.0	43.7	–	–	–	–	42.2	42.6	45.2	61.7	54.9	50.5	50.8
Married, spouse present	69.9	69.7	70.2	68.7	69.5	70.9	66.3	71.4	75.0	67.9	71.3	53.5	61.6
Northeast	36.0	36.0	35.8	34.5	36.4	37.8	37.9	40.2	40.9	28.7	24.0	30.7	16.9
Midwest	20.6	26.4	20.5	25.4	20.6	27.6	36.1	12.6	15.1	12.6	15.8	12.4	8.9
West	23.2	20.9	23.0	21.3	23.4	20.4	11.1	27.1	27.2	29.6	33.8	11.1	22.4
South	20.3	16.8	20.7	18.8	19.7	14.3	14.9	20.1	16.8	29.1	26.4	45.8	51.9
Survey year 2006	22.6	24.4	22.1	24.1	23.2	24.8	21.9	21.2	24.6	23.1	21.7	20.8	21.9
Survey year 2007	23.9	26.2	24.3	26.0	23.4	26.5	23.9	25.7	22.7	24.9	23.9	23.5	24.2
Survey year 2008	27.3	25.1	27.1	24.2	27.6	26.2	29.6	27.3	26.0	26.3	26.5	27.3	26.2
Survey year 2009	26.2	24.3	26.5	25.7	25.7	22.4	24.6	25.8	26.8	25.7	28.0	28.4	27.8
Belarus	3.5	3.4	3.4	3.1	3.7	3.8	–	–	–	–	–	–	–
Bosnia and Herzegovina	3.4	5.3	3.4	4.5	3.5	6.5	–	–	–	–	–	–	–
Bulgaria	6.4	5.0	6.5	4.5	6.2	5.8	–	–	–	–	–	–	–
Poland	13.9	20.3	14.3	20.7	13.3	19.9	–	–	–	–	–	–	–
Romania	10.4	6.1	9.9	6.0	11.2	6.2	–	–	–	–	–	–	–
Russia	30.4	23.4	31.1	26.0	29.4	20.1	–	–	–	–	–	–	–
Ukraine	17.8	20.9	17.4	20.5	18.3	21.5	–	–	–	–	–	–	–
Other countries	14.2	15.4	14.1	14.9	14.3	16.2	–	–	–	–	–	–	–
English proficiency							–	–	–	–	–	–	–
Do not speak English	.5	3.6	.6	2.3	.4	5.2	.9	.4	.5	.0	.4	.2	2.7
Speak English, but not well	4.5	20.2	4.5	18.2	4.5	22.7	4.8	5.4	4.9	.5	4.5	1.6	11.0
Speak English well	26.1	36.6	25.3	35.6	27.1	37.9	20.5	31.4	34.5	6.8	24.1	10.4	27.8
Speak English very well	59.6	33.5	60.0	36.9	59.0	29.0	63.3	54.9	54.4	44.4	63.7	50.6	53.6
Speak only English	9.4	6.2	9.6	7.0	9.1	5.3	10.5	7.9	5.8	48.2	7.3	37.2	4.9
Education													
Associate’s degree	8.7	27.2	10.4	27.2	6.6	27.3	15.5	5.9	8.4	7.9	4.6	18.6	12.6
Bachelor’s degree	38.0	49.1	40.2	49.1	35.3	49.2	35.4	34.9	40.8	39.4	44.6	46.9	51.6
Master’s degree	34.9	19.8	34.2	20.1	35.8	19.4	38.5	36.6	35.4	31.4	35.1	22.3	21.6
Professional degree	7.5	3.1	7.8	3.2	7.0	3.0	4.4	7.3	7.4	6.8	6.4	7.3	9.9
Ph.D.	10.9	.8	7.4	.5	15.3	1.2	6.2	15.3	8.0	14.6	9.3	5.1	4.3
Occupation													
Managers	21.8	–	20.0	–	23.9	–	27.0	17.6	20.4	36.0	19.4	19.9	33.5
Business and finance	11.9	–	15.2	–	7.8	–	12.2	11.5	13.3	9.8	10.9	12.6	11.3
Computer	16.0	–	9.0	–	24.9	–	10.2	20.5	19.6	8.9	24.3	7.1	5.3
Engineering	7.8	–	3.8	–	12.9	–	7.9	6.8	7.7	6.8	7.9	5.1	6.4
Sciences	5.6	–	5.7	–	5.5	–	5.2	7.0	4.5	7.2	6.1	3.1	3.6
Social services	2.3	–	3.0	–	1.4	–	3.0	1.7	2.8	1.5	1.5	7.4	4.0
Law	1.7	–	2.3	–	1.1	–	1.7	1.6	1.6	1.8	.8	1.4	1.9
Education	13.7	–	16.9	–	9.7	–	12.6	14.6	11.2	13.8	11.0	14.1	17.7
Entertainment	6.0	–	6.6	–	5.3	–	7.6	6.2	4.7	7.6	2.9	2.6	6.4
Healthcare	13.1	–	17.6	–	7.5	–	12.8	12.5	14.3	6.6	15.3	26.8	10.0
Other white collar	–	30.7	–	41.0	–	17.3	–	–	–	–	–	–	–
Service	–	41.8	–	48.2	–	33.5	–	–	–	–	–	–	–
Manual worker	–	26.3	–	8.9	–	48.6	–	–	–	–	–	–	–
Unemployed	–	1.3	–	1.9	–	.5	–	–	–	–	–	–	–
Personal income (mean)	60,527	30,613	47,660	23,507	76,913	39,755	51,840	62,655	62,647	94,420	63,307	50,430	49,062
$0 or less	4.2	6.9	6.6	10.3	1.2	2.6	4.8	5.5	2.7	4.5	4.7	3.1	5.4
$1 - $9999	6.3	12.8	8.7	17.3	3.4	7.1	6.9	6.6	7.6	5.4	6.2	5.7	7.3
$10,000 - $19,999	6.9	17.9	8.9	21.5	4.3	13.2	6.6	7.1	7.1	5.1	7.1	8.2	11.3
$20,000–49,999	32.5	46.8	37.2	42.8	26.7	52.0	38.6	26.8	29.0	21.2	25.7	4.2	40.9
$50,000 - $79,999	26.0	10.9	24.4	5.7	28.1	17.5	26.6	26.1	27.5	20.0	26.8	26.1	20.4
$80,000 - $99,000	9.8	2.1	6.7	1.1	13.8	3.5	6.8	11.6	12.5	10.5	12.4	7.2	5.4
$100,000 or higher	14.2	2.6	7.6	1.4	22.7	4.1	9.9	16.4	13.6	33.2	17.2	7.6	9.3
Self-employed	9.5	13.5	7.7	10.8	11.7	16.8	10.2	10.0	9.0	10.0	6.1	4.9	10.7
Naturalized Citizen	54.4	45.0	56.6	45.0	51.7	45.0	45.0	56.9	69.1	14.4	28.3	40.9	29.8
Country-Level Variables													
GDP per capita <$5000	46.4	48.1	45.2	44.4	48.1	52.9	–	–	–	–	–	–	–
Free countries	40.0	39.6	40.0	40.1	40.0	39.0	–	–	–	–	–	–	–
Partly free countries	26.1	33.6	25.5	30.8	26.9	37.1	–	–	–	–	–	–	–
Not free countries	33.9	26.9	34.5	29.1	33.2	23.9	–	–	–	–	–	–	–
N	8871	5699	5045	3312	3826	2387	1178	2697	1602	9428	51,337	6544	13,143
N weighted	188,523	133,678	105,598	75,215	82,925	58,463	26,167	57,254	33,589	181,692	1,143,777	167,502	295,363

Eastern European immigrants without professional or managerial occupations have a slightly shorter average length of stay in the U.S. (13 years), tend to be older (41 years), and are more likely to reside in the Midwest (26%) than immigrants holding professional or managerial occupations. They are also more likely to immigrate from Poland and Ukraine and less likely from Russia, Bulgaria and Romania.

### Education

As can be seen in Table 3, the most prevalent educational category among Eastern European professionals is a bachelor’s degree (38%) followed by a master’s degree (35%) and a Ph.D. (11%). In comparison, Eastern European skilled immigrants without professional or managerial occupations are more likely to hold an associate’s (27%) or a bachelor’s degree (49%) and less likely to hold a master’s degree (20%), professional degree (3%), or a Ph.D. (1%). These differences confirm the importance of human capital in socioeconomic adaptation of immigrant professionals with immigrants holding advanced educational degrees having a higher occupational status than less educated immigrants (Bodankin & Semyonov, 2016).

Eastern Europeans in professional or managerial occupations have a higher share of professional or Ph.D. degrees (18%) than Asians (16%), blacks (12%), or Hispanics (14%), and lag only somewhat behind professional immigrants from Northern and Western Europe (21%) in this respect. Among the top three immigrant-sending countries, Russian professionals are by far the most likely to have a professional degree or a Ph.D. (23%), followed by their Ukrainian (15%) and Polish counterparts (11%). About 91% of Eastern European professionals have a bachelor’s degree or higher, a pattern comparable to Asian and other European immigrants (95 and 92%, respectively), and higher than the share of equally educated black (82%) and Hispanic immigrants (87%). Gender differences in educational attainment show that among professionals from Eastern Europe, women are more likely to hold an associate’s degree or a bachelor’s degree, but less likely to hold a Ph.D. degree than men. The share of professional and master degree holders is very similar for both genders.

### Occupation

Table 3 also shows that the majority of Eastern European professionals are managers (22%) followed by computer professionals (16%), educators (14%), and healthcare professionals (13%). These findings are consistent with the literature that suggests immigrant professionals in the U.S. tend to be concentrated in certain occupations, including computer-related and medical professions (Batalova & Lowell, 2007). Their counterparts without professional or managerial occupations tend to concentrate in service occupations (42%) followed by other white-collar jobs (31%), manual jobs (26%), or are unemployed (1%).

Gender differences in occupational concentrations suggest that Eastern European professional women are predominantly healthcare workers or educators and men tend to hold managerial or computer-related occupations. Management, computers, education, and healthcare are preferred occupational areas of Polish, Russian, and Ukrainian professionals. Eastern European professionals are occupationally similar to Asians, who also tend to be concentrated in computer-related occupations, management, healthcare, and education. These occupations are also the most prevalent among black, Hispanic, and other European immigrants in addition to business and finance, which indicates limited variations in occupational backgrounds across different groups of immigrant professionals.

Examining specific occupations within broader occupational categories, Table 4 shows that accounting/audit is the most prevalent occupation among new Eastern European professionals. This is also the most common occupation among Polish and Ukrainian professionals while Russian immigrants tend to be computer software engineers.

**Table 4:** Top five occupational categories of post-1991 immigrant professionals from Eastern Europe and various foreign-born groups, 2006–2009 ACS

All EE	EE men	EE women	Polish	Russian	Ukrainian	Northern and Western Europe	Asian	Black	Hispanic
Accountants and Auditors (6.7%)	Computer software engineers (10.9%)	Accountants/auditors (9%)	Accountants/auditors (6.8%)	Computer software engineers (8.3%)	Accountants/auditors (7.8%)	Misc. managers including postmasters/ superintend. (8.2%)	Computer software engineers (12.2%)	Registered nurses (15.3%)	Elementary and middle school teachers (5.8%)
Computer software engineers (6.2%)	Postsecond. teachers (6.7%)	Registered nurses (6.7%)	Registered nurses (5.1%)	Accountants/auditors (6.6%)	Computer software engineers (7.6%)	Postsecond. teachers (7.1%)	Postsecond. teachers (6.6%)	Accountants and Auditors (7.1%)	Miscellaneous managers (4.9%)
Postsecond. teachers (5.8%)	Computer programmers (6.1%)	Postsecond. teachers (5.1%)	Postsecond. teachers (4.4%)	Postsecond. teachers (6.5%)	Computer programmers (6.4%)	Chief executives and legislators (3.9%)	Registered nurses (6.4%)	Elementary and middle school teachers (4.7%)	Accountants and auditors (4.8%)
Computer programmers (4.5%)	Misc. managers including postmasters/superintendents (5.3%)	Physicians and surgeons (4.0%)	Misc. managers including postmasters/superintendents (4.2%)	Computer programmers (6.2%)	Registered nurses (5.3)	Computer software engineers (3.5%)	Accountants and auditors (5.7%)	Postsecondary teachers (4.4%)	Postsecond. teachers (4.5%)
Registered nurses (4.2%)	Accountants/auditors (3.8%)	Elementary and middle school teachers (3.3%)	Designers (4.0%)	Registered nurses (4.0%)	Postsecondary teachers (3.8%)	Accountants/ auditors; Marketing and sales managers (3.2%)	Computer scientists and system analysts (4.6%)	Miscellaneous managers (3.4%)	First line supervisors/managers of retail sales workers (3.7%)

As presented in Table 4, Eastern European men tend to be computer software engineers while women work predominantly as accountants and auditors. Other occupational categories also point to clear gender differences in occupational concentrations. Eastern European women are likely to be registered nurses, physicians/surgeons, and elementary school teachers while men are represented primarily among computer software engineers, programmers, and managers. Eastern European women are the only immigrant group with physicians and surgeons among the top-five occupational categories. Polish are the only group with a high prevalence of designers, while immigrants from Northern and Western Europe are unique in their high likelihood of being chief executives and legislators.

Table 5 illustrates the relationship between educational attainment and professionalization. The results indicate that Eastern European Ph.D. holders are the most likely to be professionals or managers (95%), while the likelihood is the lowest among immigrants with an associate’s degree (31%). This pattern is consistent for other groups, which suggests that having more education increases occupational status among skilled immigrants (Bodankin & Semyonov, 2016). Among immigrants with professional degrees or Ph.D.’s, there is no gender difference in the likelihood of holding professional/managerial occupations; at lower educational levels, Eastern European women are more likely to be professionals or managers than men. Table 5 also shows that Eastern European immigrants may experience more difficulties in transferring their human capital than other immigrant groups. Eastern European skilled immigrants are less likely to hold professional or managerial occupations than all other groups except Hispanics. About 59% of Eastern Europeans with at least an associate’s degree hold professional or managerial occupations, lower that the corresponding percentage for black (62%), Asian (73%), and Northern/Western European immigrants (78%). This pattern exists at all educational levels with the smallest gap among Ph.D. holders.

**Table 5:** The likelihood of holding a professional/managerial occupation among post-1991 Eastern European immigrants and various immigrant groups in each educational category (percent), 2006–2009 ACS

	All EE	EE women	EE men	Polish	Russian	Ukrainian	N/W Europe	Asian	Black	Hispanic
Associate’s degree or higher	58.5	58.4	58.7	49.1	64.6	54.5	77.5	73.2	61.9	51.4
N	14,570	8357	6213	2203	4037	2844	25,877	136,383	24,886	58,288
N weighted	322,201	180,813	141,388	53,345	88,573	61,582	488,438	2,926,317	625,062	1,363,904
Associate’s degree	31.1	35.0	25.4	30.2	35.6	24.0	54.8	44.8	42.4	35.4
N	2370	1435	935	508	438	569	4132	15,244	6489	14,900
N weighted	52,813	31,424	21,389	13,446	9508	11,728	76,023	330,672	166,652	360,935
Bachelor’s degree	52.2	53.5	50.4	46.6	54.3	51.7	73.7	67.4	61.3	50.0
N	5917	3489	2428	781	1557	1162	10,540	68,488	11,206	28,641
N weighted	137,339	79,348	57,991	19,906	36,793	26,514	204,696	1,488,397	290,541	685,174
Master’s degree	71.3	70.5	72.4	60.0	74.8	70.8	86.5	87.2	79.8	75.1
N	4284	2464	1820	740	1310	796	6739	33,995	4980	8855
N weighted	92,249	51,215	41,034	16,785	27,972	16,799	126,034	731,148	118,107	193,262
Professional degree	77.3	77.4	77.1	74.2	83.8	70.1	90.5	91.6	85.2	62.7
N	881	531	350	86	245	165	1766	9123	1286	4320
N weighted	18,238	10,677	7561	1566	4999	3526	32,504	183,259	29,153	91,457
Ph.D.	94.9	95.2	94.8	98.5	94.3	89.6	96.4	96.4	92.0	86.5
N	1118	438	680	88	487	152	2700	9533	925	1572
N weighted	21,562	8149	13,413	1642	9301	3015	49,181	192,841	20,609	33,076

A series of logistic regression equations were estimated to predict the likelihood of holding a professional or managerial job among Eastern European skilled immigrants (Table 6). As expected, immigrants who have been residing in the U.S. for a longer period of time and are married with their spouse present in the U.S. are more likely to be professionals or managers. Contradicting the initial hypothesis, the likelihood of professionalization decreases with age, perhaps because older immigrants are more likely to experience language barriers, discrimination, have out-of-date skills, or are unable to transfer foreign credentials than younger immigrants. English proficiency and education increase the likelihood of holding professional or managerial jobs among educated immigrants while being self-employed decreases the odds. Gender difference in the likelihood of professionalization is not significant. This result corresponds to earlier discussed findings which suggest that better educated women have lower occupational status than men, noting that the pattern is reversed at lower educational levels. Region is not a significant predictor of occupational attainment, with an exception of immigrants residing in the Midwest who are less likely than immigrants in the Northeast to be professionals or managers. Expectedly, country of origin appears to affect the likelihood of professionalization with immigrants from Bosnia and Herzegovina and Poland having a lower likelihood of holding professional or managerial jobs. In comparison, immigrants from Belarus, Romania, and Russia are more likely to be professionals or managers than their counterparts from Ukraine. Country-level predictors did not have a significant effect on the likelihood of professionalization.

**Table 6:** Logistic regression estimates predicting the probability of having a professional/managerial occupation among post-1991 Eastern European immigrants with an associate’s degree or higher, 2006–2009 ACS

Predictor	Model 1		Model 2		Model 3		Model 4	
	B	Odds ratio	B	Odds ratio	B	Odds ratio	B	Odds ratio
Length of stay	.067*** (.009)	1.069	.068*** (.009)	1.071	.066*** (.009)	1.069	.065*** (.010)	1.068
Age	−.025*** (.005)	.975	−.027*** (.004)	.973	−.025*** (.005)	.975	−.026*** (.005)	.974
Male	−.013 (.059)	.987	−.005 (.056)	.995	−.011 (.053)	.989	.002 (.057)	1.002
Married, spouse present	.101** (.030)	1.107	.094** (.029)	1.098	.102** (.030)	1.108	.104** (.034)	1.110
U.S. Region (ref. = Northeast)								
Midwest	−.113* (.077)	.893	−.061* (.073)	.941	−.113 (.076)	.893	−.085 (.072)	.918
West	.165 (.095)	1.179	.111 (.085)	1.118	.166 (.091)	1.181	.158 (.097)	1.171
South	.083 (.090)	1.087	.058 (.074)	1.059	.085 (.084)	1.089	.091 (.088)	1.095
Survey year (ref. = 2006)								
Survey year 2007	−.048 (.080)	.954	−.059 (.080)	.943	−.048 (.080)	.953	−.055 (.079)	.946
Survey year 2008	.215* (.097)	1.240	.206* (.096)	1.228	.214* (.098)	1.239	.209* (.098)	1.233
Survey year 2009	.171* (.070)	1.186	.166* (.070)	1.180	.171* (.070)	1.186	.168* (.069)	1.183
English proficiency	.528*** (.065)	1.695	.522*** (.064)	1.686	.528*** (.063)	1.696	.535*** (.065)	1.707
Education	.807*** (.032)	2.241	.785*** (.030)	2.193	.806*** (.028)	2.240	.793*** (.028)	2.210
Self-employment	−.358*** (.045)	.699	−.357*** (.046)	.700	−.361*** (.052)	.697	−.365*** (.046)	.694
Country of origin (ref. = Ukraine)								
Belarus			.168*** (.015)	1.183				
Bosnia/Herzegovina			−.202*** (.035)	.817				
Bulgaria			.048 (.032)	1.049				
Poland			−.265*** (.021)	.767				
Romania			.371*** (.034)	1.450				
Russia			.241*** (.006)	1.273				
Other			−.046 (.115)	.956				
GDP per capita <$5000					−.023 (.148)	.977		
Personal freedom (ref. = free countries)								
Partly free							−.074 (.157)	.928
Not free							.234 (.145)	1.263
Constant	−12.251***		−11.911***		−12.234***		−12.112***	
-2log likelihood	365,680.07		363,335.35		365,672.02		364,734.34	
Model χ^2^	71,604.04***		73,948.76***		71,612.09***		72,549.77***	
Pseudo R^2^	.27		.28		.27		.27	
Degrees of freedom	13		20		14		15	
N N before weighting	322,201 14,570		322,201 14,570		322,201 14,570		322,201 14,570	

*p < .05 **p < .01 ***p < .001 (two-tailed test)

### Income

Income is an important measure of the overall socioeconomic status of immigrants in the United States (Portes & Rumbaut, 2014). Even among highly educated immigrants, a high amount of human capital leads to better socioeconomic adaptation only if immigrants are able to earn sufficient income (Portes & Rumbaut, 2001). The results in Table 3 point to an income gap between Eastern European professionals and other equally educated immigrant groups. The average personal income of Eastern European professionals is $60,527. On average, Eastern European professionals earn a higher income than black ($50,430) and Hispanic immigrants ($49,062), but their income is lower than the income of Asian professionals ($63,307) as well as professionals from Northern and Western Europe ($94,420). The percentage distributions across various income categories further confirm the income disadvantage of Eastern European professionals who are less likely to earn $80,000 or more (24%) or $100,000 or more (14%) than Asians (30% and 17%) and other European professionals (44% and 32%). These findings suggest a clear hierarchy of human capital to income conversion, and are consistent with the prior literature suggesting that professionals from countries outside Northern and Western Europe or North America experience more difficulties in transferring their credentials and continuing their careers in the host country (Guo, 2009; Reitz, 2001; Slade, 2015). The literature partially attributed this discrepancy to non-recognition of foreign educational credentials, which indicates that income of immigrant professionals depends on country or region of origin and where immigrants received their education.

The results also reveal a gender wage gap of almost $30,000 (Table 3), which can be partially attributed to the above discussed disparities in occupational statuses with female professionals being less represented in high-paying occupations than their male counterparts.

Eastern European skilled immigrants without professional or managerial occupations earn significantly less than their counterparts who are professionals or managers (Table 3). The gap of almost $30,000 suggests an income disadvantage experienced by skilled immigrants without professional or managerial occupations. Regardless of the immigrants’ occupational status, the most prevalent income category for Eastern European skilled immigrants is $20,000 to $49,999.

Table 7 shows that incomes of Eastern European professionals vary depending on occupational category. The highest paid are professionals in computer occupations ($78,606), followed by healthcare workers ($73,785) and engineers ($67,227). The lowest paid professionals are social service workers ($30,982). Among males, the healthcare workers tend to have the highest income ($106,164). Among females, computer professionals earn the most ($63,431). Similarly to the pooled sample, Russian and Ukrainian professionals in computer occupations earn the highest income. Among Polish professionals, healthcare workers earn the highest incomes.

**Table 7:** Mean personal income of post-1991 immigrant professionals from Eastern Europe and various foreign-born groups, 2006–2009 ACS

	All EE	EE men	EE women	Polish	Russian	Ukrainian	Northern and Western Europe	Asian	Black	Hispanic
All occupations	60,527	76,913	47,660	51,840	62,655	62,647	94,420	63,307	50,430	49,062
Management	65,009	81,480	49,563	49,062	70,413	69,186	125,424	69,621	51,350	57,046
Business/Finance	57,069	77,480	48,820	63,070	58,637	58,321	105,242	56,614	51,307	47,767
Computer	78,606	85,617	63,431	63,712	80,616	84,268	96,960	73,794	57,072	59,815
Engineering	67,227	73,458	50,398	57,627	69,972	66,262	95,127	70,828	54,798	55,577
Science	56,617	68,848	47,395	49,539	61,367	54,404	75,993	53,355	52,255	53,379
Education	33,518	45,587	28,053	31,389	34,721	28,542	42,404	29,214	35,480	28,748
Healthcare	73,785	106,164	63,010	71,197	71,370	70,948	78,581	75,351	60,068	62,003
Social Services	30,982	34,927	29,510	26,125	30,477	33,460	39,222	29,695	32,472	33,014
Law	64,666	103,460	50,251	38,520	71,704	68,788	116,068	63,151	65,146	53,143
Entertainment	40,888	53,621	32,820	37,662	36,763	29,383	61,834	41,988	34,861	36,792

Eastern European professionals in all occupational categories tend to have substantially lower incomes than skilled workers from Northern and Western Europe despite their similar educational backgrounds. The disparity appears to be the greatest in managerial occupations where the income gap is $60,415. A large income gap of $48,173 also exists among business and finance professionals. The income difference between Eastern European professionals and their Northern and Western European counterparts is the lowest among social service workers and educators. Even in these occupations, the latter group tends to earn incomes higher by more than $8000. Wide income disparities across occupational categories exist also between Northern and Western European professionals and other immigrant groups, especially when compared to black and Hispanic immigrants.

Table 8 shows cross-country differences in the average personal income. Consistent with the literature (Bodankin & Semyonov, 2016; Guo, 2009; Slade, 2015), it appears that disparities in earnings exist depending on the immigrants’ country of origin with the income ranging from $78,198 among professionals from Croatia to $49,527 among Albanian professionals. Immigrants from the Czech Republic ranked second in average income after Croatian professionals ($72,670) followed by Hungarian ($68,338) and Romanian immigrants ($65,267). Comparing income rankings with rankings on education reveals that among Eastern European immigrants, being ranked high on education does not necessarily correlate with high income rankings. For example, Slovak immigrants have the highest share of skilled immigrants with a master’s degree or higher (71%), but they ranked 14th in income. Similarly, Bulgarian and Polish immigrants have a higher share of immigrants with graduate degrees compared to other countries (65% and 50%, respectively), but these groups ranked low on income. On the other hand, Croatian immigrants had the highest income, but they ranked 8th in their share of immigrants with at least a master’s degree (50%).

**Table 8:** Socioeconomic profiles of post-1991 immigrant professionals from Eastern Europe by country of origin, 2006–2009 ACS

Country	Percent with Master’s degree or higher	Most prevalent occupational category	Average personal income	N/N weighted
Albania	33.6	Management (21.8%)	49,527	227/5072
Belarus	48.1	Computers (22.0%)	60,910	326/6641
Bosnia and Herzegovina	24.1	Management (32.5%)	53,886	279/6503
Bulgaria	64.7	Management (25.6%)	56,895	558/11,989
Croatia	49.1	Management (29.3%)	78,198	115/2240
Czech Republic	69.4	Management (21.9%)	72,670	135/2579
Hungary	54.9	Management (24.4%)	68,338	227/4364
Latvia	43.0	Management (22.7%)	64,381	91/2078
Lithuania	42.5	Management (31.2%)	60,118	165/3715
Macedonia	28.6	Management (22%)	54,374	55/1021
Moldova	44.5	Management (26.1%)	58,754	155/3047
Poland	49.1	Management (27.0%)	51,840	1178/26,167
Romania	56.7	Management (19.8%)	65,267	936/19,688
Russia	59.2	Computers (20.5%)	62,655	2697/57,254
Slovakia	71.3	Management (23.4%)	53,233	125/2576
Ukraine	50.8	Management (20.4%)	62,647	1602/33,589

The OLS regression was used to examine the determinants of income among Eastern European skilled immigrants regardless of their occupational status (Table 9) and also among immigrants who hold professional or managerial occupations (results not shown because predictors had the same effect on income regardless of the immigrants’ occupational status). The dependent variable was log transformed, so an unstandardized regression coefficient can be interpreted as a percentage change in the dependent variable for a one-unit change in the independent variable after multiplying it by 100.

**Table 9:** Estimates of OLS regression models predicting logged personal income of post-1991 immigrants from Eastern Europe with an associate’s degree or higher, 2006–2009 ACS

Predictor	Model 1		Model 2		Model 3		Model 4	
	B	β	B	β	B	β	B	β
Constant	3.144***		3.134***		3.142***		3.160***	
Length of stay	.022*** (.001)	.217	.023*** (.001)	.224	.022*** (.001)	.217	.022*** (.001)	.221
Age	.001* (.000)	.027	.001* (.001)	.027	.001* (.000)	.027	.001** (.001)	.030
Male	.219*** (.008)	.238	.217*** (.008)	.236	.218*** (.008)	.238	.218*** (.008)	.238
Married, spouse present	.008 (.009)	.008	.009 (.009)	.009	.008 (.009)	.008	.010 (.009)	.010
U.S. Region (ref. = Northeast)								
Midwest	−.028* (.011)	−.025	−.035** (.011)	−.032	−.028* (.011)	−.025	−.035** (.011)	−.032
West	−.013 (.011)	−.012	−.016 (.011)	−.015	−.013 (.011)	−.012	−.012 (.011)	−.011
South	−.044*** (.011)	−.038	−.051*** (.011)	−.043	−.044*** (.011)	−.038	−.046*** (.011)	−.039
Survey year (ref. = 2006)								
Survey year 2007	.026* (.012)	.025	.026* (.012)	.024	.026* (.012)	.025	.026* (.012)	.025
Survey year 2008	.066*** (.012)	.064	.066*** (.012)	.064	.066*** (.012)	.064	.066*** (.012)	.064
Survey year 2009	.074*** (.012)	.070	.074*** (.012)	.071	.074*** (.012)	.070	.075*** (.012)	.071
English proficiency	.050*** (.006)	.093	.045*** (.006)	.084	.050*** (.006)	.093	.048*** (.006)	.089
Education	.047*** (.004)	.107	.047*** (.004)	.107	.047*** (.004)	.107	.047*** (.004)	.107
Professional/Managerial occupation	.198*** (.010)	.213	.198*** (.010)	.212	.198*** (.010)	.213	.199*** (.010)	.214
Self-employed	−.091*** (.017)	−.063	−.092*** (.017)	−.064	−.091*** (.017)	−.063	−.094*** (.017)	−.065
Country of origin (ref. = Ukraine)								
Belarus			−.016 (.023)	−.006				
Bosnia/Herzegovina			.034 (.018)	.015				
Bulgaria			.045* (.019)	.023				
Poland			.018 (.014)	.015				
Romania			.078*** (.016)	.048				
Russia			.011 (.012)	.011				
Other			.032* (.014)	.024				
GDP per capita <$5000					.003 (.008)	.003		
Personal freedom (ref. = free countries)								
Partly free							−.035*** (.010)	−.035
Not free							−.034*** (.010)	−.034
R^2^ (adjusted)	.230		.233		.230		.232	
F	6521.66***		4402.27***		6087.17***		5746.52***	
N	305,010		305,010		305,010		305,010	
N before weighting	13,741		13,741		13,741		13,741	

*p < .05 **p < .01 ***p < .001 (two-tailed test)

As expected, for each additional year of stay, the predicted income of skilled immigrants increases by 2%. Age is positively associated with income, but only increases earnings by less than 1% for each year increase in age. Expectedly, the men’s income is about 22% higher than the women’s among immigrants with professional occupations and 20% higher if immigrants hold non-professional jobs. The effect of marital status on income is not significant regardless of the immigrants’ occupational status.

Immigrants residing in all regions tend to earn less than immigrants in the Northeast, but the difference between those in the West and those in the Northeast is not significant. English proficiency, education, and holding a professional or managerial position increase income among skilled immigrants. Respondents surveyed in all the years tend to have higher personal incomes than those surveyed in 2006.

With the exception of the immigrants from Bulgaria, Romania, and other countries combined who tend to earn higher incomes than immigrants from Ukraine, the cross-country differences in income are not significant. The differences in income, depending on countries’ GDP per capita, also did not reach statistical significance; nonetheless, results are in agreement with the expectation that immigrants from countries classified as partly free or not free tend to earn less than those from free countries, indicating that unfavorable political conditions negatively affect income of skilled immigrants.

## Discussion and conclusion

The goal of this study was to examine the socioeconomic adaptation of post-1991 skilled immigrants from Eastern Europe in the United States. The study focused on immigrants from sixteen Eastern European countries who immigrated to the U.S. after the dissolution of the Soviet Union. The minimum educational level of immigrants included in the analysis was an associate’s degree. The experiences of immigrants with and without professional or managerial occupations were compared to better ascertain differences in socioeconomic outcomes.

Economic incorporation of immigrant professionals can be evaluated and discussed in light of several alternative theoretical frameworks. According to the classical assimilation perspective, all skilled immigrants are expected to gradually adapt socioeconomically over time. The segmented assimilation theory proposes differences in socioeconomic adaptation depending on the individual or group characteristics. The findings presented in this study support the latter perspective, revealing cross-group and cross-country differences in socioeconomic adaptation of immigrant professionals as well as differences depending on selected individual and country-level characteristics.

Overall, the Eastern European immigrants with professional and managerial occupations are highly educated and they are educationally similar to skilled immigrants from Asia and Northern/Western Europe; despite educational and occupational similarities, the results reveal disparities in income, especially between Eastern European professionals and their counterparts from Northern and Western Europe. The results confirm that there are wide variations in earnings experienced by immigrant professionals (Bodankin & Semyonov, 2016) with all groups in the analysis having lower incomes than Northern and Western European origin groups.

Eastern European immigrants with at least an associate’s degree have a lower likelihood of employment in professional or managerial occupations than all other groups, except Hispanics. Over 40% of educated Eastern European immigrants do not have a professional or managerial job. These immigrants experience lower occupational status and, correspondingly, lower incomes. Eastern Europeans have the lowest share of associate and master degree holders employed as professionals or managers. This suggests that Eastern European skilled immigrants experience more difficulties in transferring their human capital and continuing their careers than other groups in the analysis, especially immigrants from Northern and Western Europe.

The results indicate that an education-occupation mismatch experienced by post-1991 Eastern European immigrants in general also exists among Eastern European professionals. The socioeconomic disparities experienced by Eastern European professionals can perhaps be attributed to differences in their educational systems, pre-emigration labor market experiences, and the language barrier, which may hinder the transfer of skills and human capital into the U.S. labor market. These disparities also suggest that despite their white racial background, Eastern European professionals experience an income disadvantage similar to non-white immigrants.

The results further reveal that socioeconomic adaptation of Eastern European professionals is segmented by the immigrants’ country of origin. Consistent with the theory of segmented assimilation, results show major socioeconomic differences across Eastern European groups. Among the top three Eastern European countries, a clear hierarchy emerges with Russian professionals doing the best socioeconomically followed by immigrants from Ukraine and Poland. Russian professionals have the highest share of Ph.D. holders and Russian and Ukrainian immigrants have the highest share of professional degree holders. Russians also have the highest share of professionals among immigrants with an associate’s degree or higher followed by Ukrainian and Polish immigrants. Russian and Ukrainian professionals have the same average income, but the average income of Polish immigrants is lower, further highlighting segmented adaptation trajectories that correspond to cross-country differences in educational attainment and occupational concentrations.

Eastern European skilled women are as likely to hold professional or managerial jobs as their male counterparts, but despite similar likelihood of professionalization, men and women differ in their occupational concentrations and income. Women are more concentrated in business, finance, healthcare, and education, while men tend to be employed in computer-related occupations and engineering. A significant gender-wage gap suggests that women may be concentrated in lower-paying jobs. Professional women from Eastern Europe earn on average $30,000 less than their male counterparts. The gap persists across all professional occupations and it is the largest in law and healthcare.

Supporting the theory of segmented assimilation, post-1991 Eastern European skilled immigrants tend to adapt socioeconomically in different ways depending on their individual and country-level characteristics. Immigrants who reside in the United States for a longer period of time tend to adapt better socioeconomically than their respective counterparts. Older skilled immigrants tend to earn higher incomes, but they have a lower likelihood of finding a professional career than younger workers. Married immigrants are more likely to be professionals, but marriage and presence of spouse has no effect on income. English proficiency, higher education, and higher occupational status are characteristics associated with better socioeconomic adaptation among Eastern European skilled immigrants. There are no consistent differences in adaptation across different regions. Immigrant professionals from Russia and Romania tend to fare better socioeconomically and immigrants from Bosnia and Herzegovina and Poland tend to do worse than their Ukrainian counterparts. Country-level characteristics have no impact on occupational outcomes, but political conditions in the immigrants’ countries of origin negatively affect income.

There are many possible directions for further research on professionals from Eastern Europe. Future studies could examine other determinants of socioeconomic adaptation; for example, the effect of class of admission and context of reception. Other adaptation dimensions should also be studied, including cultural, structural, or political adaptation, to provide a comprehensive picture of immigrant professionals and their experiences. A qualitative component would enhance our understanding of the immigrants’ experiences. Future research should also separately compare the experiences of various Eastern European immigrant groups to better understand cross-group differences in socioeconomic adaptation.
